# Thyroid Hormone Enhances Nitric Oxide-Mediated Bacterial Clearance and Promotes Survival after Meningococcal Infection

**DOI:** 10.1371/journal.pone.0041445

**Published:** 2012-07-23

**Authors:** Yao Chen, Mikael Sjölinder, Xiao Wang, Georg Altenbacher, Matthias Hagner, Pernilla Berglund, Yumin Gao, Ting Lu, Ann-Beth Jonsson, Hong Sjölinder

**Affiliations:** Department of Genetics, Microbiology and Toxicology, Stockholm University, Stockholm, Sweden; Indian Institute of Science, India

## Abstract

Euthyroid sick syndrome characterized by reduced levels of thyroid hormones (THs) is observed in patients with meningococcal shock. It has been found that the level of THs reflects disease severity and is predictive for mortality. The present study was conducted to investigate the impact of THs on host defense during meningococcal infection. We found that supplementation of thyroxine to mice infected with *Neisseria meningitidis* enhanced bacterial clearance, attenuated the inflammatory responses and promoted survival. *In vitro* studies with macrophages revealed that THs enhanced bacteria-cell interaction and intracellular killing of meningococci by stimulating inducible nitric oxide synthase (iNos)-mediated NO production. TH treatment did not activate expression of TH receptors in macrophages. Instead, the observed TH-directed actions were mediated through nongenomic pathways involving the protein kinases PI3K and ERK1/2 and initiated at the membrane receptor integrin αvβ3. Inhibition of nongenomic TH signaling prevented iNos induction, NO production and subsequent intracellular bacterial killing by macrophages. These data demonstrate a beneficial role of THs in macrophage-mediated *N. meningitidis* clearance. TH replacement might be a novel option to control meningococcal septicemia.

## Introduction

Thyroid hormones (THs) are produced by the thyroid gland. The major form of THs in the blood is thyroxine (T4), which is then deiodinated in peripheral tissues to the active triiodothyronine (T3). THs are required for normal function of most tissues by regulating metabolism, development and differentiation [1]. T3 and T4 are lipophilic substances and are able to traverse cell membranes by passive uptake and via cell membrane transporters [2], [3]. Many cellular actions of THs are mediated by nuclear TH receptors (TRs), which are ligand-dependent transcription factors that preferentially bind T3. Two TR genes, α and β, encode multiple isoforms that can be generated by alternative splicing or promoter choice. After binding with T3 as well as a specific DNA sequence, e.g. TH response elements (TREs), in the promoter region of target genes, TRs regulate gene transcription by interacting with either co-activator or co-repressor complexes [4]. This classical genomic model of TH action has a considerable latency with response times in hours to days [5], [6]. TH-dependent signal transduction can also be initiated at the plasma membrane or in the cytoplasm. This nuclear TR-independent nongenomic action can occur within a rapid time frame of only a few minutes [7], [8]. Integrin αvβ3, a heterodimeric plasma membrane protein, contains binding domains for both T3 and T4 [9]. Study of the binding kinetics between THs and integrin αvβ3 has suggested two hormone-binding sites on the integrin αvβ3. One site binds T3 exclusively and the signal is transduced via the PI3K pathway. Another site binds to both T3 and T4 and the signal is transduced through the ERK1/2 pathway [10].

Stable TH levels are maintained and tightly regulated by thyroid-stimulating hormone (TSH). However, altered physiological and pathological conditions can affect levels of THs. The levels of THs were found to be decreased in critically ill patients, especially patients with septic shock. This condition is collectively called euthyroid sick syndrome or nonthyroidal illness syndrome [11]. Decreased level of THs is highly correlated with the severity of illness and is a powerful predictor of high mortality in critically ill patients [12], [13].

Several studies indicate that THs play a role in immune modulation. T3 stimulates keratinocyte proliferation and is necessary for optimal wound healing [14], [15]. In dendritic cells, T3 enhanced maturation and cytokine production through NF-κB-dependent TRβ1 expression [16]. Patients with hypothyroidism displayed suppressed lymphocyte function [17]. Both T3 and T4 have been shown to play a physiological role in cellular defense mechanisms by stimulating free-radical production in polymorphonuclear leucocytes (PMN) [18]. Moreover, T4 inhibits proinflammatory activity of macrophage migration inhibitory factor (MIF) [19]. Supplementation of THs exerted a beneficial effect on sepsis induced by cecal ligation and puncture in animal models [19], [20], but a beneficial role of THs in counteracting infectious disease has not been confirmed [21].

Septicemia induced by *Neisseria meningitidis* is one of the most severe infectious syndromes characterized by a sudden onset and rapid progression of disease. The euthyroid sick syndrome has been observed in patients with meningococcal shock. In both children and adult patients the levels of total T3 reflected disease severity and was predictive for mortality [22], [23]. In a previous study using a mouse disease model, we found that meningococci accumulated in the thyroid gland during sepsis and decreased level of THs was associated with the severity of disease [24]. However, little is known about the impact of THs on host defense during meningococcal infection. It remains to be clarified whether the hormonal abnormalities are determining factors in the outcome of meningococcal disease or merely represent a beneficial adaptive response to the septic condition, and if TH supplementation might have effects on meningococcal sepsis criteria.

In this study, we investigated the impact of THs on controlling meningococcal infection. Using a mouse model of meningococcal disease, we found that TH supplementation enhanced survival and attenuated meningococcal septicemia as well as inflammatory responses. Bacteria-cell interaction and intracellular killing of meningococci by macrophages was enhanced upon TH treatment. We demonstrated that TH enhanced inducible nitric oxide synthase (iNos)-mediated NO production through pathways involving PI3K and ERK1/2 and this action was initiated at the membrane receptor integrin αvβ3.

## Materials and Methods

### Ethics Statement

Mice experiments described in the present study were conducted at the animal facility of Stockholm University. The animals were handled according to directives and guidelines of the Swedish Animal Protection Agency. The study was performed under approval of the Stockholm North Ethical Committee on Animal Experiments (Approval ID: N380/08).

**Table 1:** Primer sequences used for real-time PCR.

Gene	Primer sequence (5′-3′sss)
mTRa	F: GCTGCTGATGAAGGTGACTG
	R: AAAGACCTCCAGGAAGAGTGG
mTRb	F: ACAGAAAATGGCCTTCCAGC
	R: TCTTGCTGTCATCCAGCACC
mGAPDH	F: CAACTTTGTCAAGCTCATTTCCTG
	R: CCTCTCTTGCTCAGTGTCCTT
hiNOS	F: CCTCGGCTCCAGCATGTAC
	R: TGGGACAGCTTCTGATCAATG
hIL-6	F: GGCACTGGCAGAAAACAACC
	R: GCAAGTCTCCTCATTGAATCC
hTNFα	F: CCTGCCCCAATCCCTTTATT
	R: CCCTAAGCCCCCAATTCTCT
hMIF	F: GAACCGCTCCTACAGCAAGCT
	R: GCGAAGGTGGAGTTGTTCCA
hRPL37A	F: ATTGAAATCAGCCAGCACGC
	R: AGGAACCACAGTGCCAGATCC

**Figure 1:**
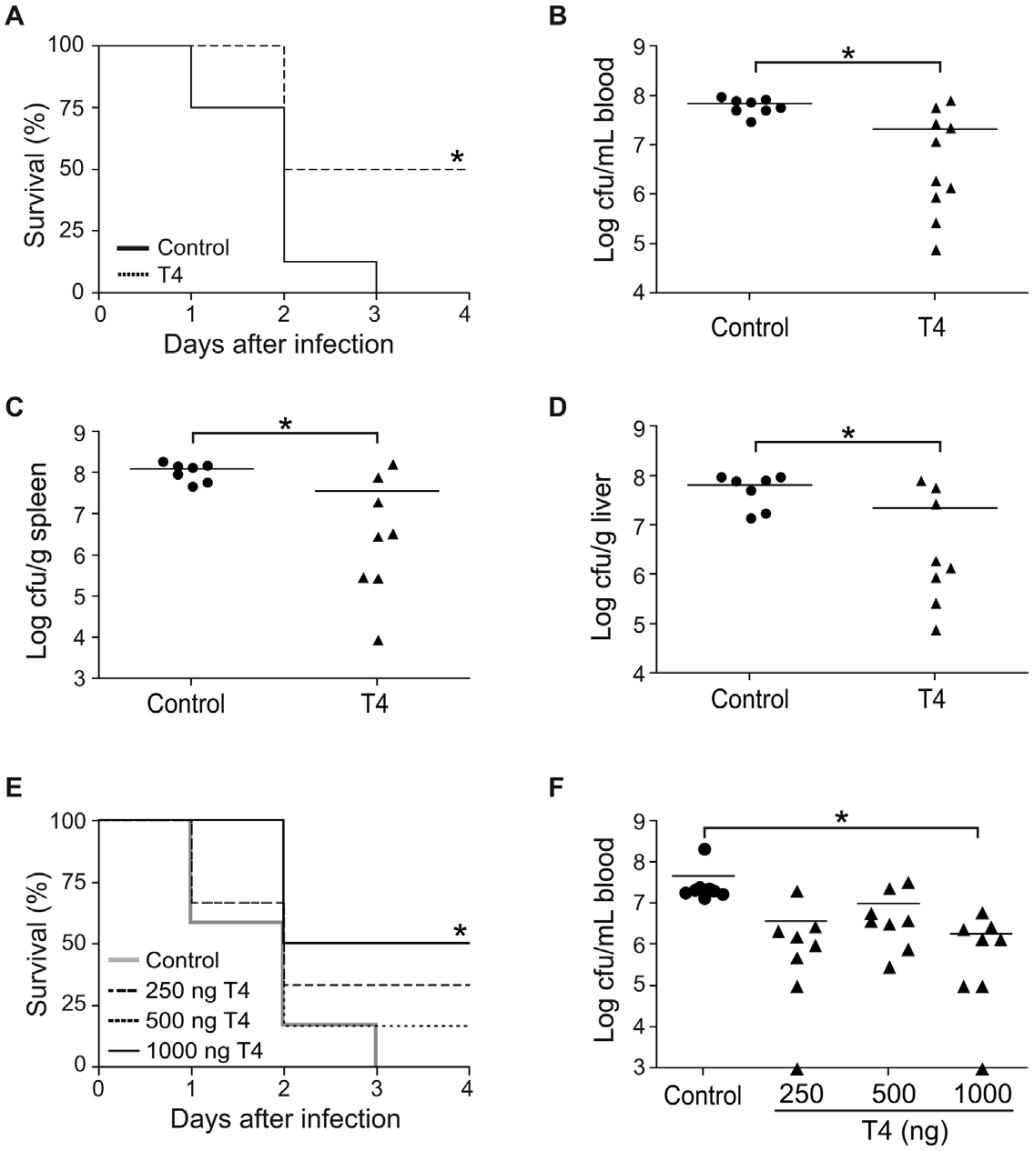
T4 enhances survival during meningococcal infection. (A-D) Mice (n = 10–12 per group) were treated with T4 (500 ng) or vehicle (control) for three days. On the second day of treatment, mice were challenged i.p. with 10^8^ CFU of the *N. meningitidis* strain FAM20. (A) Survival of T4-treated and control mice after bacterial infection. Bacterial counts in blood (B), spleen (C) and liver (D) of mice at 24 h p.i. were determined. (E–F) Mice were challenged with 10^8^ CFU of bacteria and treated with T4 (250–1000 ng/mouse) at 4 h and 24 h p.i. The control group was treated with vehicle. (E) Survival and (F) bacterial counts in blood of mice at 24 h p.i. were measured. *, *P*<0.05 (Nonparametric Mann-Whitney test in A and E, Student’s *t*-test in B–D, F). Symbols represent individual mice and the horizontal lines represent mean of the values.

**Figure 2:**
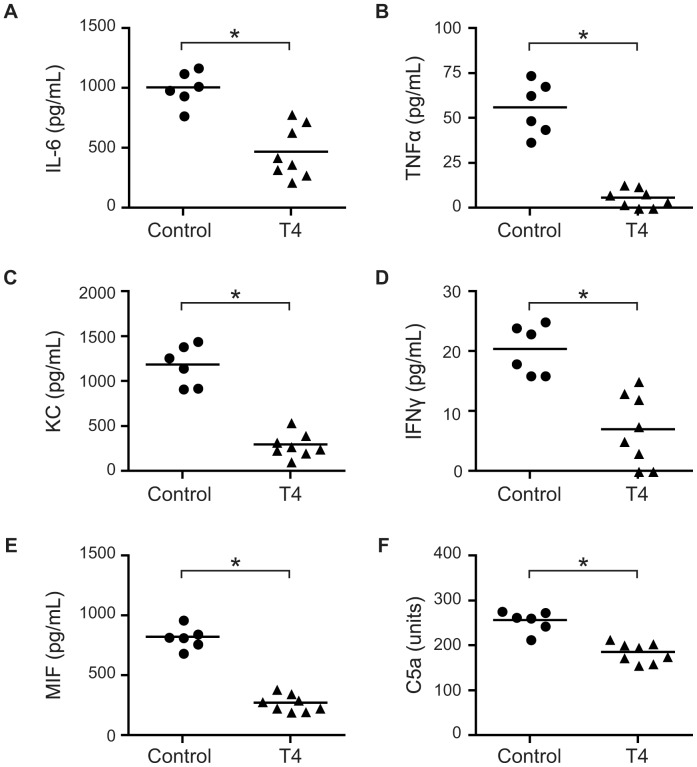
T4 attenuates inflammatory responses *in vivo*. Mice (n = 8 per group) were treated as described in Figure 1 and the concentrations of IL-6 (A), TNFα (B), KC (C), IFNγ (D), MIF (E) and C5a (F) in serum at 24 h p.i. were measured by ELISA. Symbols represent individual mice and the horizontal lines represent mean of the values. *, *P*<0.05 (Student’s *t*-test).

### Chemicals

LY294002, PD98059, Tetraiodothyroacetic acid, (S)-methylisothiourea, LPS, synthetic T3 and T4 were purchased from Sigma. MTT (3-(4,5-dimethylthiazol-2-yl)-2,5-diphenyltetrazolium bromide) was purchased from Invitrogen. To prepare stock solutions, T3 and T4 (10 mg/mL) were dissolved in 1 M NaOH; LY294002 (10 mM), PD98059 (10 mM) and tetraiodothyroacetic acid (50 mM) were dissolved in DMSO; (S)-Methylisothiourea (2 mM) was dissolved in H_2_O; MTT (5 mg/mL) and LPS (2 mg/mL) was dissolved in PBS.

**Figure 3:**
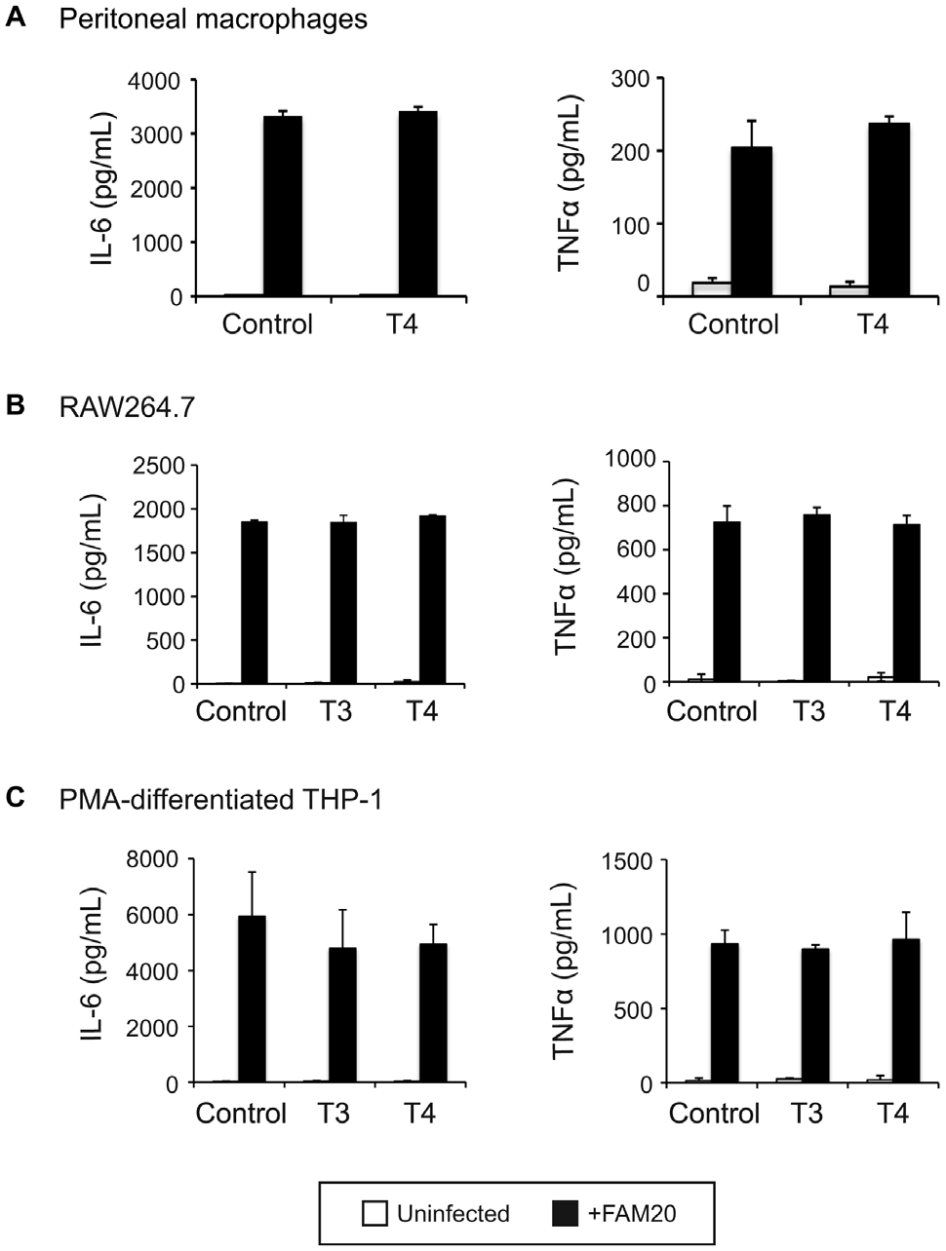
Impact of THs on macrophage cytokine production. (A) Peritoneal macrophages were collected from mice treated with T4 or vehicle as described in Figure 1. (B) RAW264.7 cells and (C) PMA-differentiated THP-1 cells were treated with 100 nM T3 or 1 µM T4 for 24 h, cells treated with vehicle NaOH were set as control. Cells were infected with FAM20 at a MOI of 200 for 24 h and levels of IL-6 and TNFα in the supernatant were quantified by ELISA. The experiments were performed in triplicate and data are presented as means ±SD.

**Figure 4:**
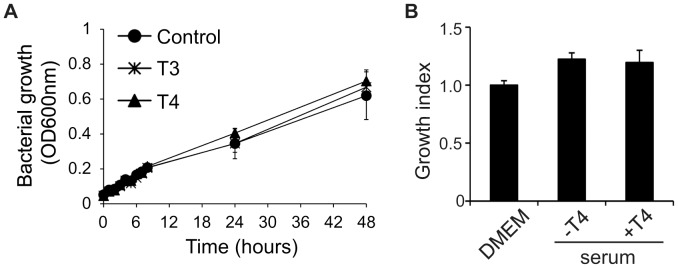
Impact of thyroid hormones on *N. meningitidis* growth. (A) Effect of THs on meningococcal growth in GC liquid. FAM20 were suspended in GC liquid containing 100 nM T3 or 1 µM T4 and allowed to grow for up to 48 h. At indicated time points, the growth of bacteria was monitored by optical measurement at 600 nm. Bacteria grown in GC liquid were set as a control. (B) Effect of THs on meningococcal growth in serum. Serum was collected from mice treated with T4 or vehicle as described in Figure 1 and mixed with FAM20. As a control, DMEM was mixed with bacteria in the same way. The bacterial solutions were incubated for 1 h at 37°C, plated after serial dilutions and the number of surviving bacteria was counted the next day. Bacterial growth index was defined as (CFU after incubation)/(CFU before incubation) and bacterial growth in DMEM was set as 1. Experiments were performed in triplicate and data are presented as means ±SD.

### Bacterial Strain

*N. meningitidis* strain FAM20 was grown for 18 h at 37°C in a 5% CO_2_ atmosphere on GC agar (Difco) supplemented with Kelloggs [25]. To prepare heat-killed bacteria, bacteria were collected from the GC agar plate, suspended in cell culture medium and heated at 65°C for 1 h.

**Figure 5:**
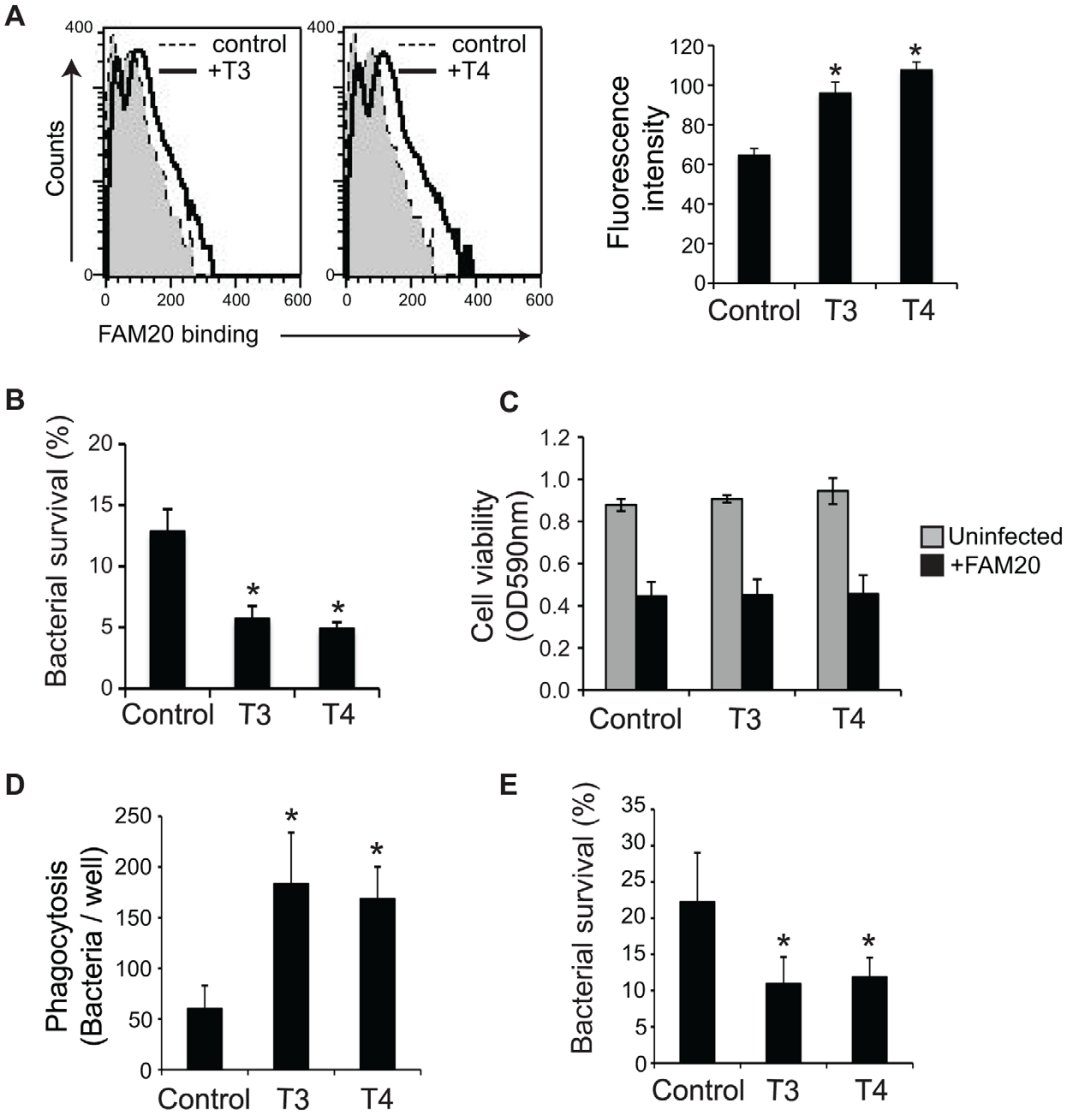
TH enhances the bactericidal activity of macrophages. (A)–(C) RAW264.7 cells were treated with 100 nM T3 or 1 µM T4 for 24 h. (A) Cells were infected with FAM20 (MOI  = 200) for 1 h at 4°C. Binding of the bacteria was determined by flow cytometry after staining with an anti-*N. meningitidis* Ab followed with an alexa 488-conjugated IgG. Mean fluorescence intensity of the entire population is presented. (B) TH-treated RAW264.7 cells were infected with FAM20 (MOI  = 200) for 3 h and intracellular bacterial survival was determined by a gentamicin protection assay as described in Materials and Methods. (C) Viability of TH-treated cells in the presence and absence of FAM20 was measured using a MTT assay. (D)-(E) PMA-differentiated THP-1 cells were treated with 100 nM T3 or 1 µM T4 for 24 h and then infected with FAM20 (MOI  = 200) for 3 h. (D) Phagocytosed bacteria and (E) Intracellular bacterial survival was determined by a gentamicin protection assay as described in Materials and Methods. Cells treated with vehicle NaOH were set as control. All experiments were performed in triplicate and data are presented as means ±SD. *, *P*<0.05 (Student’s *t*-test).

### Cell Culture and Treatment

The murine macrophage cell line, RAW 264.7 (ATCC TIB71) was cultured in DMEM (Invitrogen), plus 10% heat-inactivated fetal calf serum (FCS) at 37°C and 5% CO_2_. THP-1 (ATCC TIB 202) was cultured in RPMI (Invitrogen), plus 10% of heat-inactivated FCS and differentiated with phorbol 12-myristate 13-acetate (PMA) for 3 days. Cells were treated with T3 (1–100 nM) or T4 (0.01–1 µM) for 24 h. In the vehicle control, the same amount of NaOH was added as to the hormone-treated cells. In certain experiments inhibitors of different signaling pathways, such as LY294002 (2.5 µM), PD98059 (10 µM), tetraiodothyroacetic acid (10 µM), and S-methylisothiourea (50 µM) were added before hormone treatment. Live or heat-killed FAM20 (for NO production analysis) was added to cells at a MOI of 200 for 24 h and cell supernatants were collected for measurements of nitrate and cytokine production. Cells were harvested for determination of mRNA levels, Western blot analysis, or immunofluorescence staining. Some cells were treated with LPS (500 ng/mL) for 24 h as a control.

**Figure 6:**
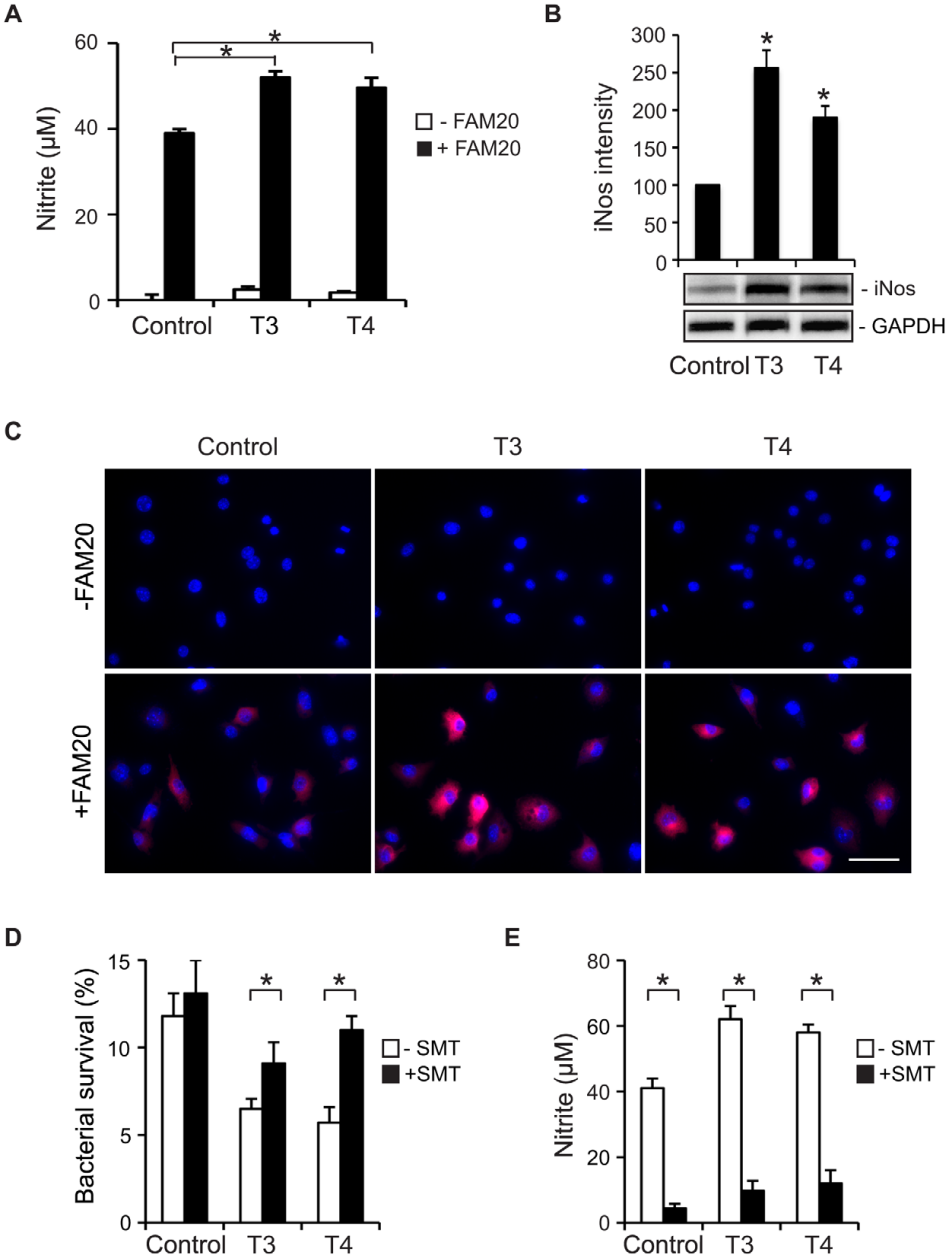
TH-enhanced bactericidal activity by macrophages is mediated through iNos/NO production. (A)–(C) RAW264.7 cells were treated with 100 nM T3 or 1 µM T4 for 24 h prior to stimulation with heat-killed *N. meningitidis* FAM20 (MOI  = 200) for 24 h. Control cells were treated with vehicle for THs. (A) Production of NO was determined by measurement of nitrite in the cell supernatant. (B) Expression of iNos protein in cells was detected by Western blot using a rabbit anti-mouse iNos Ab. Expression of GAPDH was set as a loading control. Densitometric analysis of Western blot bands was performed using Image J software and relative expression levels of iNos protein was quantified after normalization to GAPDH. Data are presented as mean ±SD of three independent experiments and are indicated as percentage of control cells, which was set as 100%. *, *P*<0.05 (Student’s *t*-test). (C) Expression of iNos in cells was visualized by immunofluorescence microscopy using an anti-iNos Ab. Cell nuclei were stained with DAPI. Representative images from three independent experiments are shown. Scale bar: 40 µm. (D)–(E) RAW264.7 cells were cultured with 100 nM T3 or 1 µM T4 in the presence of (S)-methylisothiourea (SMT) for 24 h. Vehicle for THs (NaOH) or SMT (H_2_O) was added to control or SMT-untreated cells. (D) Cells were infected with live FAM20 (MOI  = 200) and intracellular bacterial survival was determined by a gentamicin protection assay as described in Materials and Methods. (E) Cells were infected with heat-killed FAM20 (MOI  = 200) for 24 h and nitrite in the cell supernatant was determined by a Griess assay. Experiments were performed in triplicate and data are presented as means ±SD. *, *P*<0.05 (Student’s *t*-test).

### Mouse Model of Infection

The hCD46Ge transgenic mouse line was created using B6C3F1 hybrids. It carries the complete human CD46 gene and expresses CD46 in a human-like pattern [26]. CD46 play an important role in regulating complement activity and the adaptive immune response [27], [28]. In addition, both *in vitro* studies and crystal structure analysis indicated that CD46 could be used as a cellular receptor by bacteria [29], [30], [31]. Although contradicting data exist [32], [33] and the *in vivo* role of CD46 in meningococcal infection is still not absolutely clear, previous studies have shown that typical meningococcal disease can be induced in this mouse model [24], [34], [35].

**Figure 7:**
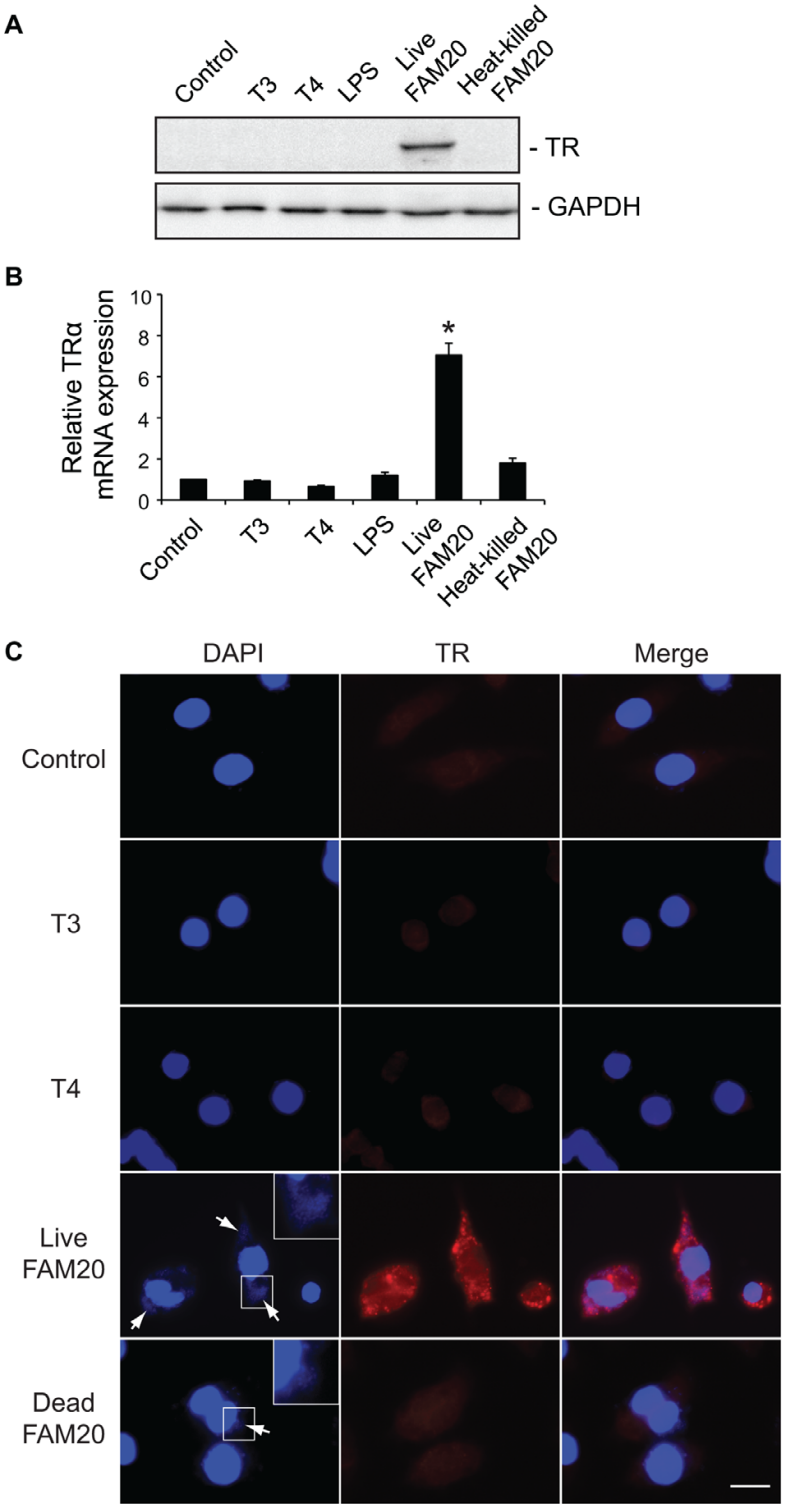
Expression of TRα in macrophages is induced by live *N. meningitidis*. RAW264.7 cells were treated with T3 (100 nM), T4 (1 µM), LPS (500 ng/ml), live FAM20 (MOI  = 2) or heat-killed FAM20 (MOI  = 200) for 24 h. (A) Expression of TRα in cells was detected by Western blot using an anti-TRα/β Ab. Expression of GAPDH was measured as a loading control. (B) Levels of TRα mRNA were analyzed by real-time PCR. Expression levels were normalized to a reference mRNA (GAPDH). The experiment was performed in triplicate and results are presented as means ±SD. *, *P*<0.05 (Student’s *t*-test). (C) Expression and localization of TR in macrophages was visualized by immunofluorescence staining using an anti-TRα/β Ab. Cell nuclei and bacteria (arrows) were stained with DAPI. Representative images are shown from experiments that were repeated three times. Scale bar: 10 µm.

Mice were challenged intraperitoneally (i.p.) with 10^8^ FAM20 suspended in 100 µL PBS. Starting one day before bacterial infection, each mouse was given i.p. 500 ng of T4 once per day for three days. In some mice, T4 (250–1000 ng) was only given at 4 h and 24 h post FAM20 infection. Control mice were injected with the same volume of vehicle in PBS. In survival studies, the health status of all mice was closely monitored for 10 days. Experiments were performed with 6–8 week old mice (n = 10–12 mice per group) and repeated two times.

**Figure 8:**
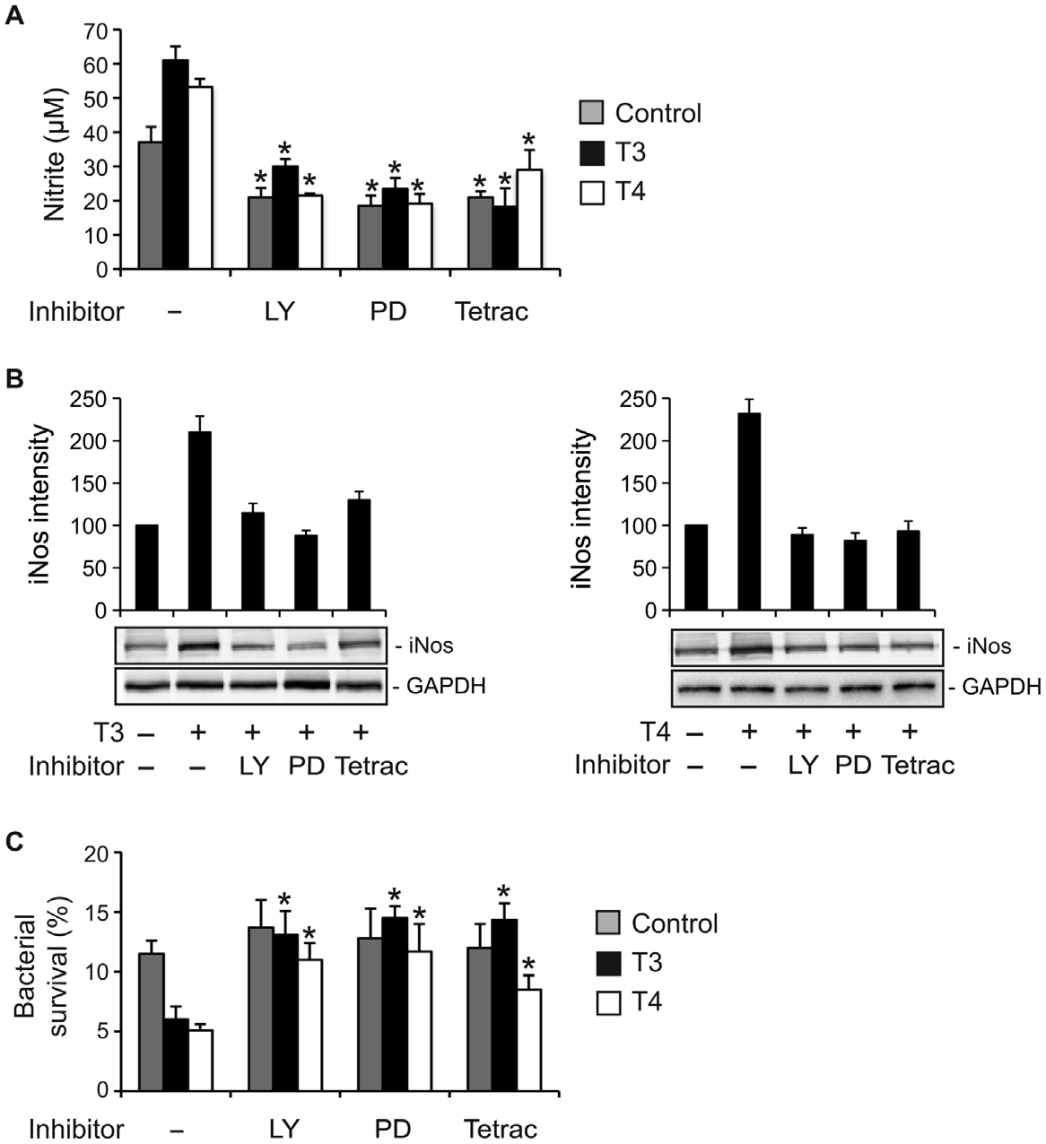
TR-independent pathways are involved in **TH-enhanced NO production and iNos expression.** RAW264.7 cells were treated with or without (Control) 100 nM T3 or 1 µM T4 for 24 h in the presence or absence of 2.5 µM LY294002 (LY, PI3K inhibitor), 10 µM PD98059 (PD, ERK1/2 inhibitor) or 10 µM tetraiodothyroacetic acid (Tetrac, T3/T4 analog to integrin αvβ3 receptor). Vehicle for THs (NaOH) or inhibitors (DMSO) was added to control or inhibitor-untreated cells. (A) Cells were infected with heat-killed FAM20 (MOI  = 200) for 24 h. Levels of nitrite in the supernatants were determined by a Griess assay. *, *P*<0.05 (Student’s *t*-test, compared to cells without inhibitor treatment). (B) Cells were infected with heat-killed FAM20 (MOI  = 200) for 24 h. Expression of iNos protein in cells was detected by Western blot using an anti-iNos Ab. Densitometry of Western blot bands was performed using Image J software and relative expression level of iNos protein is presented after normalization to GAPDH. Data is displayed as arbitrary units set to 100 for the control without TH treatment. (C) RAW264.7 cells were treated as indicated above and infected with live FAM20 (MOI  = 200) for 3 h. Intracellular survival of meningococci was determined using a gentamicin protection assay as described in Materials and Methods. Experiments were performed in triplicate and data are presented as means ±SD. *, *P*<0.05 (Student’s *t*-test, compared to cells without inhibitor treatment).

Peritoneal macrophages were harvested from mice treated with T4 or vehicle after washing the peritoneal cavity with 5 ml of ice-cold 16% sucrose. Cells were suspended in RPMI 1640 medium containing 10% FCS supplemented with penicillin and streptomycin, and seeded in a 24-well plate for 3 h. Non-adherent cells were removed and fresh medium was added. Cells were infected with live FAM20 at a MOI of 200 for 24 h and the supernatants were collected for quantification of cytokine production.

### Detection of Meningococci in Blood, Spleen and Liver

5 µL of blood was collected from the mouse tail vein at 24 h p.i., diluted in 245 µL of GC liquid and plated on GC agar plates after serial dilutions. The limit of detection is 500 CFU/mL blood. Plates were incubated over night at 37°C, 5% CO_2_ and the number of CFUs was counted next day. To assess bacterial load in organs, spleen and liver was dissected from sacrificed mice, weighed and homogenized in PBS. Tissue suspensions were plated on GC agar plates after serial dilutions and the CFUs were counted next day.

### Growth Kinetics of *N. meningitidis* Upon TH Treatment

*N. meningitidis* collected from GC agar plates were suspended in GC or GC containing T3 (100 nM) or T4 (1 µM) to OD_600nm_ 0.05. Bacteria were allowed to grow under moderate shaking at 37°C in a 5% CO_2_ atmosphere and the growth of bacteria was monitored by measuring OD_600nm_ for two days.

To examine the impact of thyroid hormones on bacterial growth *ex vivo*, we collected serum from mice treated with T4 or vehicle. *N. meningitidis* suspensions were prepared in DMEM cell culture medium and mixed with the serum at a ratio of 1∶1 to final concentrations of 10^5^ bacteria/mL blood. Bacteria grown in cell culture medium under the same conditions was set as control. The bacterial solution was then incubated for 1 h at 37°C in a 5% CO_2_ atmosphere, serially diluted in GC liquid and spread on GC agar plates to enumerate the surviving colonies after an overnight incubation. Data are presented as growth index compared to the control.

### Measurement of NO, Cytokines, Chemokines and Thyroxine

The levels of murine IL-6, TNFα, KC and IFNγ in serum or in cell supernatants were quantified by ELISA assays (Invitrogen). MIF and C5a in mouse sera were quantified by an indirect ELISA assay with a rabbit anti-MIF antibody (Santa Cruz, sc-20121) or a rat anti-mouse C5a antibody (BD Biosciences, I52–1486). Production of NO was determined by measurement of nitrite in the cell supernatant using a Griess assay (Promega). Concentrations of T4 in mouse serum were determined using an Opticoat® Thyroxine (T4) enzyme immunoassay (EIA) kit (Biotecx Laboratories).

### MTT Assay

The cell viability was determined by a MTT assay. After seeding into 96-well plates, cells were treated with T3 (1–100 nM) or T4 (0.01–1 µM) for 24 h. In the vehicle control, the same amount of NaOH was added as to the hormone-treated cells. FAM20 at MOI 200 were added for 3 h, cells were washed with PBS and extracellular bacteria were killed by treatment with gentamicin (200 µg/mL) for 2 h. The medium was then replaced with DMEM without phenol red and MTT reagent (Invitrogen) was added at a final concentration of 500 µg/mL. The plate was incubated for 2 h at 37°C in dark and the formazan crystals in the cells were dissolved in isopropanol (150 µL/well). Absorbances at 590 nm were measured using a microplate reader.

### Western Blot Analysis

After washing twice with ice-cold PBS, cells were treated with lysis buffer containing 50 mM Tris-HCl (pH 7.6), 0.3 M Sucrose, 2 mM EDTA, 1 mM PMSF, 2 mM DTT, 20% Glycerol, 0.5% IGEPAL. The concentration was measured and equal amounts (30 µg) of protein was separated by SDS-PAGE and transferred to PVDF membrane (Santa Cruz Biotechnology). The primary antibodies used in the study were a rabbit anti-mouse iNos (1∶500, MBS301453; MyBioSource) and rabbit anti-mouse TRα/β (1∶200, FL-408, sc-772; Santa Cruz Biotechnology). Expression of GAPDH (detected by a mouse GAPDH antibody, G8795; Sigma) was used as an internal control. The second antibodies were IRDye® 680 Donkey anti-Mouse IgG or IRDye® 800CW Donkey anti-Rabbit IgG (1∶15,000, LI-COR Biosciences) and were detected by Odyssey® Infrared Imaging System (LI-COR Biosciences). Densitometric analysis was performed using Image J sofeware and the relative protein expression was quantified after normalization to GAPDH.

### Immunofluorescence Staining

Cells seeded on coverslips were fixed with 4% paraformaldehyde for 30 min at RT. After blocking with Image-iT™ FX Signal Enhancer (Invitrogen), cells were incubated with a rabbit anti-mouse iNos Ab (1∶100, MBS301453; MyBioSource) or a rabbit anti-mouse TRα/β Ab (1∶50, FL-408, sc-772; Santa Cruz Biotechnology) for 1 h at RT. Alexa Fluor 594-conjugated donkey anti-rabbit IgG (1∶100, Invitrogen) was used as secondary Ab. Slides were washed and mounted in DAPI containing Prolong gold anti-fade reagent (Invitrogen). Controls for unspecific binding by both primary and secondary antibodies were included in each staining.

### Real-time PCR

Total cell RNA was isolated using a Qiagen RNeasy mini kit. 1 µg RNA was reverse-transcribed into cDNA using Superscript™ III first-strand synthesis kit and random hexamers (Invitrogen). Real-time PCR was performed with the LightCycler 480® SYBR Green I Master kit (Roche Diagnostics) using the LightCycler® 480 Real-Time PCR system (Roche Diagnostics) to quantify mRNA expression. Primers specific for mRNA encoding mouse TRα1, TRβ1, GAPDH and human iNOS, IL-6, TNFα, MIF, RPL37A (Table 1) were used at a final concentration of 1 µM in each reaction. Thermal cycling parameters were 10 s at 95°C, 10 s at 60°C and 20 s at 72°C. Expression levels were normalized to a reference gene (mouse GAPDH or human RPL37A).

### Flow Cytometry

RAW 264.7 cells were treated with 100 nM T3 or 1 µM T4 for 24 h and infected with FAM20 (M.O.I.  = 200) for 1 h at 4°C. After washing and fixation with 4% paraformaldehyde, bacterial binding was investigated using a rabbit anti-*N. meningitidis* Ab (1∶100, United States Biological, Swampscott, MA) followed by Alexa Fluor 488-conjugated goat anti-rabbit IgG (1∶5,000; Invitrogen). Samples were run on a FACS Calibur cytometer (BD Bioscience), and the resulting data were analyzed with the CellQuest Pro software (BD Bioscience).

### Intracellular Survival of Meningococci in Macrophages

A modified gentamicin protection assay [36] was used to access bacterial survival in macrophages. In brief, cells were seeded in two 24-well plates, treated with THs for 24 h and infected with FAM20 (MOI  = 200). After incubation for 3 h, unbound bacteria were removed by washing with medium and the extracellular bacteria were killed by treating cells with 200 µg/mL gentamicin for 1 h. To determine the number of total invasive bacteria, cells in one plate were lysed with 1% saponin, the lysates were plated and the number of viable bacteria was counted on the next day. Cells in a second plate were incubated with fresh medium for another 1 h. The number of viable intracellular bacteria was determined as described above. The proportion of surviving bacteria was calculated by dividing the number of viable intracellular bacteria with the number of total invasive bacteria. All samples were tested in triplicate, and experiments were repeated at least three times.

### Statistical Analysis

Nonparametric Mann-Whitney test was used to analyze significant changes in bacteremia levels and survival rates between groups. Student’s *t*-test was used for statistical analysis of cytokine levels, NO production and TR transcription. Differences between means were considered significant if *P*≤0.05.

## Results

### TH Enhances Bacterial Clearance and Survival during Meningococcal Infection

To evaluate the role of THs in host defense against *N. meningitidis* infection, we used a mouse model of meningococcal disease [34], [35]. Mice were treated with T4 one day before infection with the *N. meningitidis* strain FAM20. T4 was used for *in vivo* studies since it is usually used in normal replacement therapies in hypothyroid patients due to its longer half-life compared with T3. Further, a prolonged effect can be obtained through the conversion of T4 to T3 in peripheral tissues. Meningococcal challenge led to lethal outcome of all mice in the control group after 3 days post infection (p.i.). Survival was significantly increased in T4-treated mice, i.e. 50% of mice survived meningococcal infection until the experiment was terminated at day 10. (Figure 1A). To assess whether the differential survival rate between the two groups was reflected in levels of septicemia, we detected the bacterial numbers in the blood stream and peripheral organs at 24 h p.i. Mice supplied with T4 displayed lower bacterial numbers in the blood (Figure 1B), spleen (Figure 1C), and liver (Figure 1D) than the control group. The levels of serum T4 at 24 h p.i. were also detected, the mean T4 concentration in control mice was 1.2±0.9 µg/dL, whereas in hormone-treated mice it was increased to 5.1±2.3 µg/dL.

To further study the protective effect of T4, mice were initially challenged with FAM20 and then treated with T4 (250–1000 ng/mouse) at 4 h and 24 h post-infection. We found that T4 treatment increased survival in a dose-dependent manner (Figure 1E). A survival rate of 50% was observed in the group treated with 1000 ng T4 whereas all mice not treated with T4 died. T4-treated mice also displayed decreased numbers of bacteria in the blood (Figure 1F).

### TH Attenuates Inflammatory Responses

The potent inflammatory responses triggered by meningococcal infection are associated with severe meningococcal sepsis symptoms. We therefore determined the levels of cytokines and chemokines in serum at 24 h post meningococcal infection. Results showed that the levels of IL-6 (Figure 2A), TNFα (Figure 2B), KC (Figure 2C) and IFNγ (Figure 2D) were significantly decreased in T4-treated mice compared to the control mice. Levels of MIF, a proinflammatory cytokine that plays a critical role in the development of sepsis [37], decreased in T4-administrated mice as compared with the control mice (Figure 2E). Levels of C5a, an anaphylatoxin generated upon activation of the complement cascade, in mouse serum were also attenuated in the TH-treated group (Figure 2F).

To investigate the impact of THs on regulation of cytokine production, peritoneal macrophages from control and T4-treated mice were collected and infected with FAM20 for 24 h. Secreted cytokines were quantified by ELISA and no significant changes in levels of IL-6 or TNFα could be detected in T4-treated cells (Figure 3A). Similar observations were made when T3- or T4-treated RAW 264.7 mouse macrophages (Figure 3B) and human PMA-differentiated THP-1 cells (Figure 3C) were analysed. Transcription of cytokine genes in PMA-differentiated THP-1 cells following TH treatment was investigated further. As shown in Figure S1, transcription of mRNA encoding IL-6, TNFα and MIF was not affected by TH treatment.

### TH Does not Affect *N. meningitidis* Growth *in vitro*

Given the decreased levels of bacteremia observed in T4-treated mice, we were interested in determining if TH could affect bacterial growth. The FAM20 strain was grown in either GC liquid or DMEM cell culture medium supplied with different amounts of T3 (1–100 nM) or T4 (0.01–1 µM) and bacterial growth was determined by measuring the absorbance at 600 nm. We found that supplementation of T3 or T4 did not affect bacterial growth at all tested concentrations (Figure 4A and data not shown). The possible role of THs in regulating bacterial growth in the bloodstream was examined further. Bacterial growth in sera collected from control and T4-treated mice was determined by viable counts after 1 h incubation. Again, TH supplementation did not affect bacterial growth in the serum (Figure 4B).

### TH Enhances Bactericidal Activity of Macrophages

Macrophages play an important role in immune defense against meningococcal infection. We investigated whether TH could affect the bactericidal activity of macrophages and consequently enhance survival in the mouse disease model following meningococcal infection. The first step of phagocytosis by macrophages, interaction between bacteria and the cells, was detected. RAW264.7 cells were treated with T3 or T4 for 24 h, and then incubated with bacteria for 1 h at 4°C since the phagocytic activity of macrophages is reduced at low temperature [38]. Flow cytometry showed that T3- or T4-treated cells could bind more bacteria than untreated cells (Figure 5A).

Macrophage-mediated intracellular killing of meningococci was further determined by a gentamicin protection assay. We found that both T3 and T4 augmented intracellular bacterial killing by RAW 264.7 cells (Figure 5B). TH can stimulate proliferation of many cell types [39], [40], [41], [42] and the impact of TH on macrophage viability was therefore studied by a MTT assay to rule out the possibility that enhanced bactericidal activity could be a result of increased cell viability. The results presented in Figure 5C showed that T3 or T4 did not affect cell viability in both the presence and absence of meningococci. Enhanced bacteria-cell interaction (Figure 5D) and intracellular killing (Figure 5E) upon T3 or T4 treatment were also observed in human THP-1 monocyte-derived macrophages. These results indicate that TH treatment enhances the bactericidal activity of macrophages.

### TH Promotes iNos-mediated NO Production in Macrophages during Meningococcal Infection

Knowing that meningococci were killed more efficiently in macrophages upon TH treatment, we next tried to determine whether NO generation in macrophages might be affected. Live meningococci have an efficient NO detoxification system and bacteria can deplete nitrite under both aerobic and microaerobic growth conditions [43]. Therefore we first tested infection conditions using live or heat-killed FAM20 with infection periods from 30 min to 72 h. Infection with either live or heat-killed FAM20 induced iNos expression in cells (data not shown). However, nitrite in the supernatants could only be detected when cells were infected with heat-killed FAM20 (Figure S2). Therefore, infection with heat-killed FAM20 for 24 h at a MOI of 200 was applied throughout the study to investigate NO production. RAW 264.7 cells were incubated with THs for 24 h prior to infection with heat-killed FAM20 and nitrite in the supernatant was measured at 24 h p.i. Both T3 and T4 could enhance NO production by macrophages. Upon meningococcal stimulation TH-treated cells generated significantly more NO compared with the control cells (Figure 6A).

In macrophages NO is generated from arginine by iNos. We therefore detected iNos expression in TH-treated cells by immunoblotting. Without bacterial stimulation, no iNos signal could be detected (data not shown). Infection with FAM20 (both heat-killed and live bacteria) stimulated iNos expression in cells and expression was further enhanced by T3 or T4 treatment (Figure 6B and data not shown). iNos expression was also visualized by immunofluorescence staining (Figure 6C). Hormone or bacterial treatment did not induce obvious changes in cell morphology. Notably, both enhanced NO production and iNos expression could only be detected after prolonged hormone treatment, i.e. at around 18 h p.i. (Figure S2 and data not shown). Treating cells with S-methylisothiourea (SMT), an iNos inhibitor, reversed T3- or T4-enhanced bactericidal effects (Figure 6D) and NO production (Figure 6E). Furthermore, thyroid hormone-enhanced iNOS transcription was detected by real-time PCR in human THP-1 monocyte-derived macrophages (Figure S3A). Enhanced intracellular bacterial survival was also observed when cells were co-treated with SMT (Figure S3B). These data support the idea that the enhanced bactericidal activity of macrophages was promoted by TH-induced iNOS expression.

### TH-induced iNos Production is not Mediated by TR

Since the observed TH-mediated effects in macrophages only could be detected after prolonged treatment and RAW264.7 cells have been demonstrated to contain TR [44], we investigated if TR might be involved in TH-induced iNos expression. RAW264.7 cells were treated with T3 or T4 for 2 to 48 h. Cell lysates were prepared and TR expression was detected by Western blot. Surprisingly, we could not detect expression of TR in cells at any time point (Figure 7A and data not shown). Treatment of the cells with either *E. coli* LPS or heat-killed FAM20, which enhanced iNos expression (Figure 6B), did not induce TR expression (Figure 7A). However, expression of TRα could be induced after 2 h infection with live *N. meningitidis* strain FAM20 (Figure 7A). LOS or capsule deleted meningococcal mutant strains showed a similar capacity to induce TRα expression (data not shown), indicating that cytotoxic effects and bacterial-cell interaction did not play a determinant role in inducing TRα expression. Induction of TRα upon stimulation with live meningococci was further confirmed by real-time PCR (Figure 7B). A nearly 7-fold increase of TRα transcription was detected when cells were infected with live FAM20 compared to cells treated with T3 or T4. Upon infection with live meningococci, induced TRα was detected in the cytoplasmic region of more than 90% of macrophages (Figure 7C). Neither cytoplasmic nor nuclear TR expression could be observed in cells treated with T3, T4 or heat-killed FAM20 (Figure 7C).

### TH-induced iNos Expression in Macrophages is Mediated by PI3K and ERK1/2 and Initiated at Integrin αvβ3

Nuclear TR-independent nongenomic action of TH through the PI3K and ERK1/2 signaling pathways has been characterized in fibroblasts [7] and myocytes [45]. We analyzed the possible role of these signaling cascades in TH-enhanced NO production by macrophages during meningococcal infection. When cells were treated with specific inhibitors of PI3K (LY294002) or ERK1/2 (PD98059), both TH-induced NO production (Figure 8A) and iNos expression (Figure 8B) were abrogated indicating that PI3K and ERK1/2 were involved in TH-mediated iNos expression, which consequently resulted in enhanced NO production.

We further explored the possible involvement of integrin αvβ3 in TH-mediated effects in macrophages since specific binding sites on this receptor for THs have been identified [9]. As shown in Figure 8A and B, NO production and iNos expression induced by T3 or T4 was impaired in the presence of tetraiodothyroacetic acid, which is a TH analog and displaces T4 and T3 from the binding domains at the integrin αvβ3. Involvement of PI3K, ERK1/2 and integrin αvβ3 in TH-enhanced iNOS production was also observed in PMA-differentiated THP-1 cells (Figure S4).

### Meningococcal Survival in Macrophages Increases when Nongenomic Pathways of TH Action are Blocked

Nitric oxide exerts direct bactericidal activity and is one of the most important bactericidal molecules preventing intracellular bacterial survival in macrophages. Since we found that TH-induced NO production in macrophages was impaired by the presence of LY294002, PD98059 or tetraiodothyroacetic acid, we wished to confirm increased intracellular survival of meningococci under these conditions. The survival status of *N. meningitidis* in macrophages was examined by a gentamicin protection assay. As expected, viability of intracellular meningococci was significantly increased when effects of T3 or T4 were blocked with inhibitors mentioned above (Figure 8C). These results are in agreement with the data showed in Figure 8A and B, where inhibition of PI3K, ERK1/2 or binding sites of THs at the integrin αvβ3 receptor impaired hormone-mediated iNos/NO production.

## Discussion

Euthyroid sick syndrome has been reported in patients with meningococcal septic shock and the levels of THs have been found to be a parameter for evaluating disease severity [22], [46]. Application of corticosteroids in sepsis has become a standard of care [47] and therapy with TH with a fairly beneficial effect has been observed in patients with cardiac disease [48] and transplantation [49]. The role of THs in meningococcal septicemia has up to now not been investigated even though THs replacement could exert a beneficial effect on preventing septic shock [19], [20]. We observed that T4 exerts beneficial effects on controlling meningococcal septicemia with improved survival in the mouse disease model. Inflammatory responses and bacterial loads in the bloodstream were attenuated with exogenous T4 administration. However, both *in vitro* and *ex vivo* data showed that THs did not affect meningococcal growth. Moreover, *in vitro* production of cytokines by macrophages was marginally affected. This observation is in line with a previous study, which showed that the cytokine expression pattern of T lymphocytes was not affected by TH levels in patients [50]. Mice responded differently to T4 treatment in this study, some hormone-treated mice died with bacterial levels in the organs that were similar to the levels observed in the control group. Supplemented and endogenous TH might act in concert to achieve critical TH levels. Secretion of TH and other hormones is characterized by pulsatility leading to high variations in hormone levels [51]. Therefore, critical TH levels might not be reached in some mice. Furthermore, high levels of proinflammatory cytokines in certain mice could also dampen the effect of hormone supplementation since the cytokine MIF can bind to T4 [19].

Although T3 or T4 did not affect macrophage viability, bacteria-cell interaction was enhanced. Phagocytosis is driven by a fine controlled remodeling of the actin cytoskeleton that takes place at the leading edge of motile cells and at adhesion sites [52], [53]. The ability of TH to influence cytoskeleton, cell migration and cell attachment has been studied in astrocytes and neurons [54], [55]. Interestingly, by using a 2D gel electrophoresis proteomics approach we also identified that TH treatment enhances the expression of several cytoskeleton-related proteins in macrophages (unpublished results). The role of TH in phagocytosis of *N. meningitidis* and other pathogens will need to be further defined.

In addition to enhanced bacteria-cell interaction, bacterial survival in hormone-treated macrophages decreased significantly compared to control cells. We demonstrated that iNos expression and consequently NO production by macrophages was induced by TH treatment. Treating cells with S-methylisothiourea, an iNos inhibitor, reversed the hormone-induced NO production and bactericidal activity in macrophages supporting the notion that TH enhanced bactericidal activity by stimulating iNos expression in macrophages and possibly other inflammatory cells. Multiple reports have described that TH can induce NO release in several cell types such as myocytes [45]. In addition, free-radical production can be induced by TH in human PMNs [56]. To our knowledge, this is the first study where TH has been shown to control meningococcal survival in macrophages by up-regulating iNos/NO production.

Expression of TRα and TRβ in RAW264.7 cells can be induced by IFNγ [44], suggesting that a direct genomic action of TH may regulate macrophage functions. One unexpected finding in this study was the observation that cytoplasmic expression of TRα in murine macrophages could be specifically induced by live meningococci and the induction could be detected as early as 2 h p.i. Neither heat-killed FAM20 nor *E. coli* LPS induced a similar response. The neisserial opacity-associated (Opa) protein, which plays an important role in bacterial adhesion to and invasion of human cells, was found to interact with the thyroid hormone regulation system by binding to both a cytoplasmic thyroid hormone receptor and the thyroid hormone receptor interacting protein 6 (TRIP6) [57]. The possible role of TR in cell response to bacterial infection will need further study.

We did not detect any TR expression in cells after TH treatment, nor could we see an altered distribution pattern of TR in the cells. Since both live and heat-killed meningococci can induce iNos, TRα expression induced by live meningococci seems not to be important for iNos induction in macrophages. It therefore appears likely that nongenomic actions may contribute to TH-induced iNos expression in macrophages. We could demonstrate a role for pathways involving PI3K and ERK1/2 since LY294002 (PI3K inhibitor) or PD98059 (ERK inhibitor) reversed TH-induced NO production and iNos expression by macrophages. Furthermore, the TH-enhanced bactericidal activity of macrophages was also reversed by treating cells with LY294002 or PD98059. Attenuated iNos expression in cells together with enhanced intracellular bacterial survival was also observed when cells were treated with tetraiodothyroacetic acid, a T3/T4 analog that blocks the binding site of T3 and T4 on integrin αvβ3, which is known to initiate nongenomic TH signaling. TH-enhanced bactericidal activities might also be complemented by TH deiodination, which has been described in leukocytes [58], [59].

Nongenomic actions of TH are usually rapid [60]. However, we observed that iNos expression induced by T3 or T4 required at least 18 h stimulation. Delayed nongenomic actions of TH have also been observed in murine tumor T lymphocytes [61] and rat liver cells [62]. The timescale of nongenomic action is likely dependent on cell type and specific functions [45]. Considering our results showing that TH does not have an obvious effect on production of inflammatory cytokines by macrophages, a chronic nongenomic action mediated by TH to induce NO might present an important mechanism used by the host to maintain immune cell homeostasis in response to bacterial-mediated immune stimulation.

In summary, we showed a beneficial role of TH in the control of experimental meningococcal septicemia. Further studies to examine effects of clinical hypothyroidism on cellular immune response may be helpful to determine whether TH supplementation, either alone or as adjunctive therapy, could be beneficial for the outcome of patients with meningococcal infection.

## Supporting Information

Figure S1 **Proinflammatory cytokine mRNA expression of in TH-treated macrophages.** PMA-differentiated human THP-1 monocytes were treated with 100 nM T3 or 1 µM T4 for 24 h prior to infection with *N. meningitidis* FAM20 at a MOI of 200 for 24 h. Control cells were treated with vehicle. Total RNA was extracted from cell lysates and the relative expression of mRNA encoding IL-6 (A), TNFα (B) and MIF (C) was analyzed by real-time PCR as described in Materials and Methods. Data were normalized to the reference gene (RPL37A) and fold increase values compared to the uninfected condition are displayed. The experiment was performed in triplicate and results are presented as means ±SD.
(TIF)

Figure S2 **Nitrite degradation by live** ***N. meningitidis*** **.** RAW264.7 cells were treated with T3 (0–100 nM) for 24 h prior to infection with vehicle (A), live FAM20 (B) or heat-killed FAM20 (C) at a MOI of 200. At indicated time points, the cell supernatant was collected and the concentration of nitrite was determined by a Griess assay. The experiment was performed in triplicate and results are presented as means ±SD.
(TIF)

Figure S3 **TH enhances iNOS production and bactericidal activity of macrophages.** PMA-differentiated human THP-1 monocytes were treated with 100 nM T3 or 1 µM T4 for 24 h. Control cells were treated with vehicle. (A) Cells were infected with *N. meningitidis* FAM20 at a MOI of 200 for 24 h and the relative expression of iNOS mRNA was analyzed by real-time PCR as described in Materials and Methods. Data were normalized to the reference gene (RPL37A) and fold increase values compared to the uninfected condition are displayed. (B) If indicated, cells were co-treated with the iNOS inhibitor SMT prior to infection with FAM20 at a MOI of 200. Intracellular bacterial survival was determined by a gentamicin protection assay as described in Materials and Methods. All experiments were performed in triplicate and results are presented as means ±SD. *, *P*<0.05 (Student’s *t*-test).
(TIF)

Figure S4 **PI3K, ERK1/2 and integrin αvβ3 are involved in TH-enhanced iNOS expression.** PMA-differentiated human THP-1 monocytes were treated with (A) 100 nM T3 or (B) 1 µM T4 for 24 h in the presence or absence of 2.5 µM LY294002 (LY, PI3K inhibitor), 10 µM PD98059 (PD, ERK1/2 inhibitor) or 10 µM tetraiodothyroacetic acid (Tetrac, T3/T4 analog to integrin αvβ3 receptor). Control cells were treated with vehicles (DMSO for inhibitors and NaOH for TH). Cells were infected with *N. meningitidis* FAM20 at a MOI of 200 for 24 h and the relative expression of iNOS was analyzed by real-time PCR. Data were normalized to the reference gene (RPL37A) and fold increase values compared to the uninfected condition are displayed. All experiments were performed in triplicate and results are presented as means ±SD. *, *P*<0.05 (Student’s *t*-test).
(TIF)
