# Supplementary figures and images for: Thyroid Hormone Enhances Nitric Oxide-Mediated Bacterial Clearance and Promotes Survival after Meningococcal Infection
Source: PLoS One. 2012 Jul 23;7(7):e41445. doi: 10.1371/journal.pone.0041445 (PMC3402396; doi:10.1371/journal.pone.0041445)

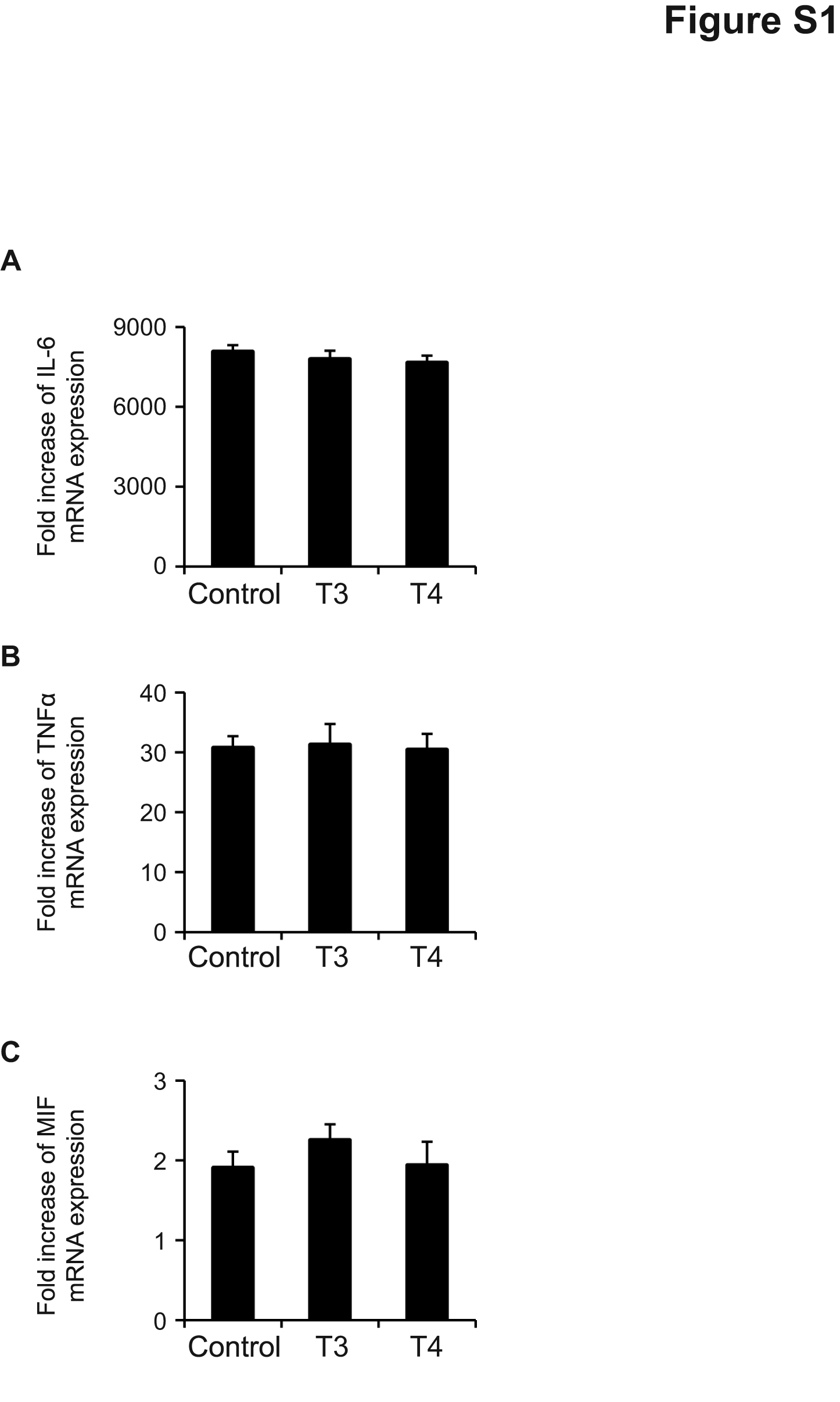

Supplement: Figure S1 — Proinflammatory cytokine mRNA expression of in TH-treated macrophages. PMA-differentiated human THP-1 monocytes were treated with 100 nM T3 or 1 µM T4 for 24 h prior to infection with N. meningitidis FAM20 at a MOI of 200 for 24 h. Control cells were treated with vehicle. Total RNA was extracted from cell lysates and the relative expression of mRNA encoding IL-6 (A), TNFα (B) and MIF (C) was analyzed by real-time PCR as described in Materials and Methods. Data were normalized to the reference gene (RPL37A) and fold increase values compared to the uninfected condition are displayed. The experiment was performed in triplicate and results are presented as means ±SD. (TIF) [file pone.0041445.s001.tif]

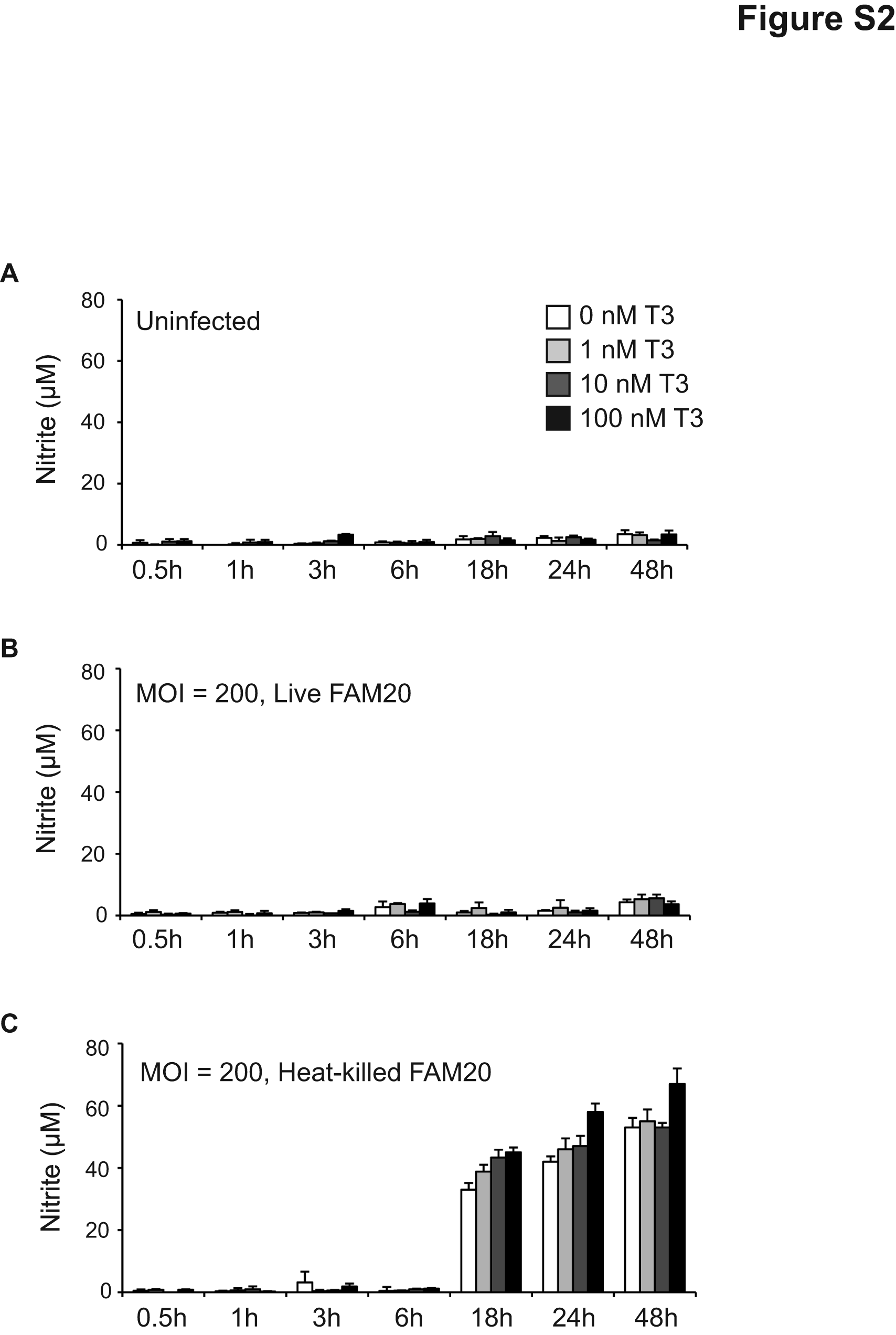

Supplement: Figure S2 — Nitrite degradation by live N. meningitidis . RAW264.7 cells were treated with T3 (0–100 nM) for 24 h prior to infection with vehicle (A), live FAM20 (B) or heat-killed FAM20 (C) at a MOI of 200. At indicated time points, the cell supernatant was collected and the concentration of nitrite was determined by a Griess assay. The experiment was performed in triplicate and results are presented as means ±SD. (TIF) [file pone.0041445.s002.tif]

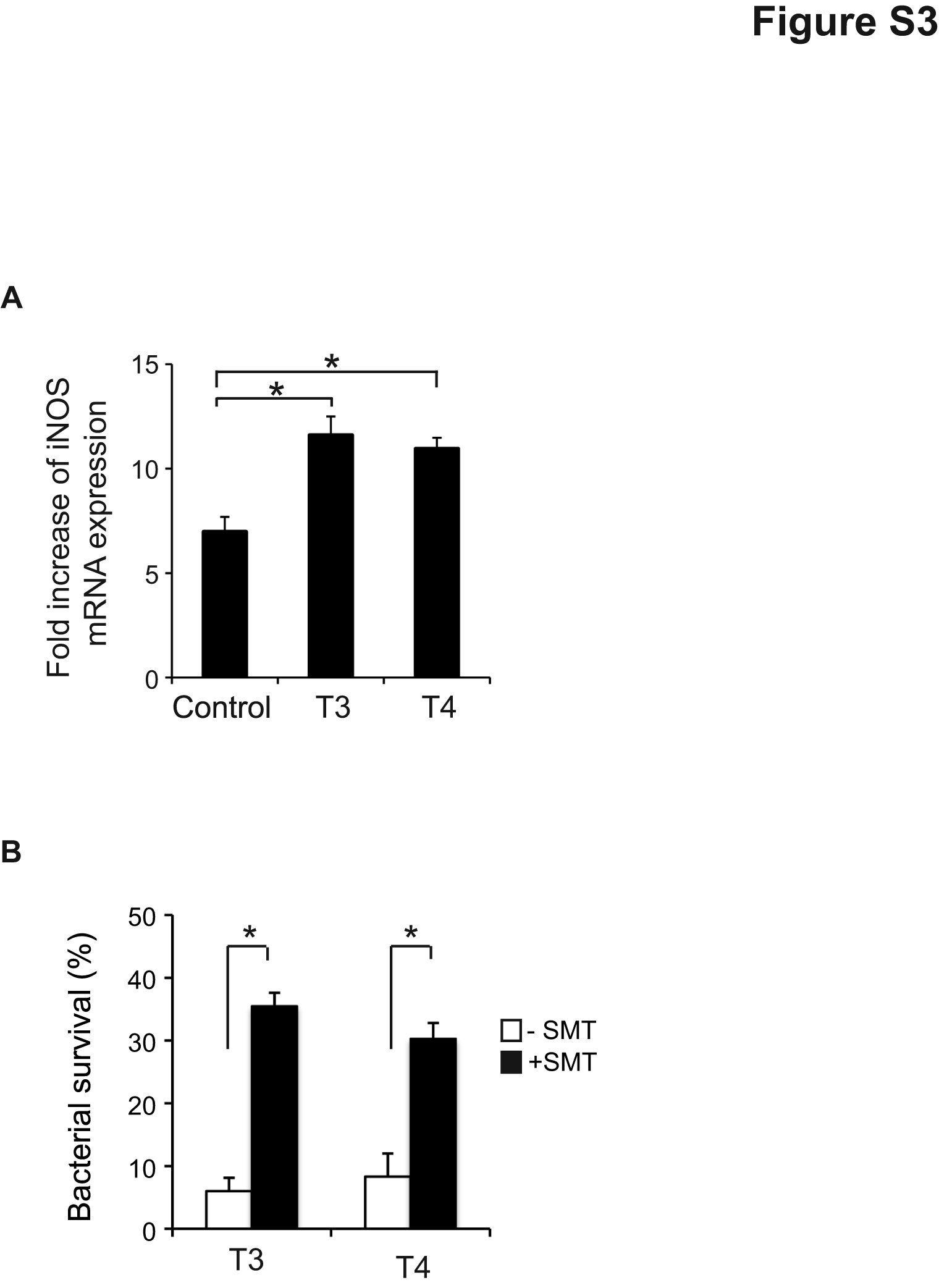

Supplement: Figure S3 — TH enhances iNOS production and bactericidal activity of macrophages. PMA-differentiated human THP-1 monocytes were treated with 100 nM T3 or 1 µM T4 for 24 h. Control cells were treated with vehicle. (A) Cells were infected with N. meningitidis FAM20 at a MOI of 200 for 24 h and the relative expression of iNOS mRNA was analyzed by real-time PCR as described in Materials and Methods. Data were normalized to the reference gene (RPL37A) and fold increase values compared to the uninfected condition are displayed. (B) If indicated, cells were co-treated with the iNOS inhibitor SMT prior to infection with FAM20 at a MOI of 200. Intracellular bacterial survival was determined by a gentamicin protection assay as described in Materials and Methods. All experiments were performed in triplicate and results are presented as means ±SD. *, P<0.05 (Student’s t-test). (TIF) [file pone.0041445.s003.tif]

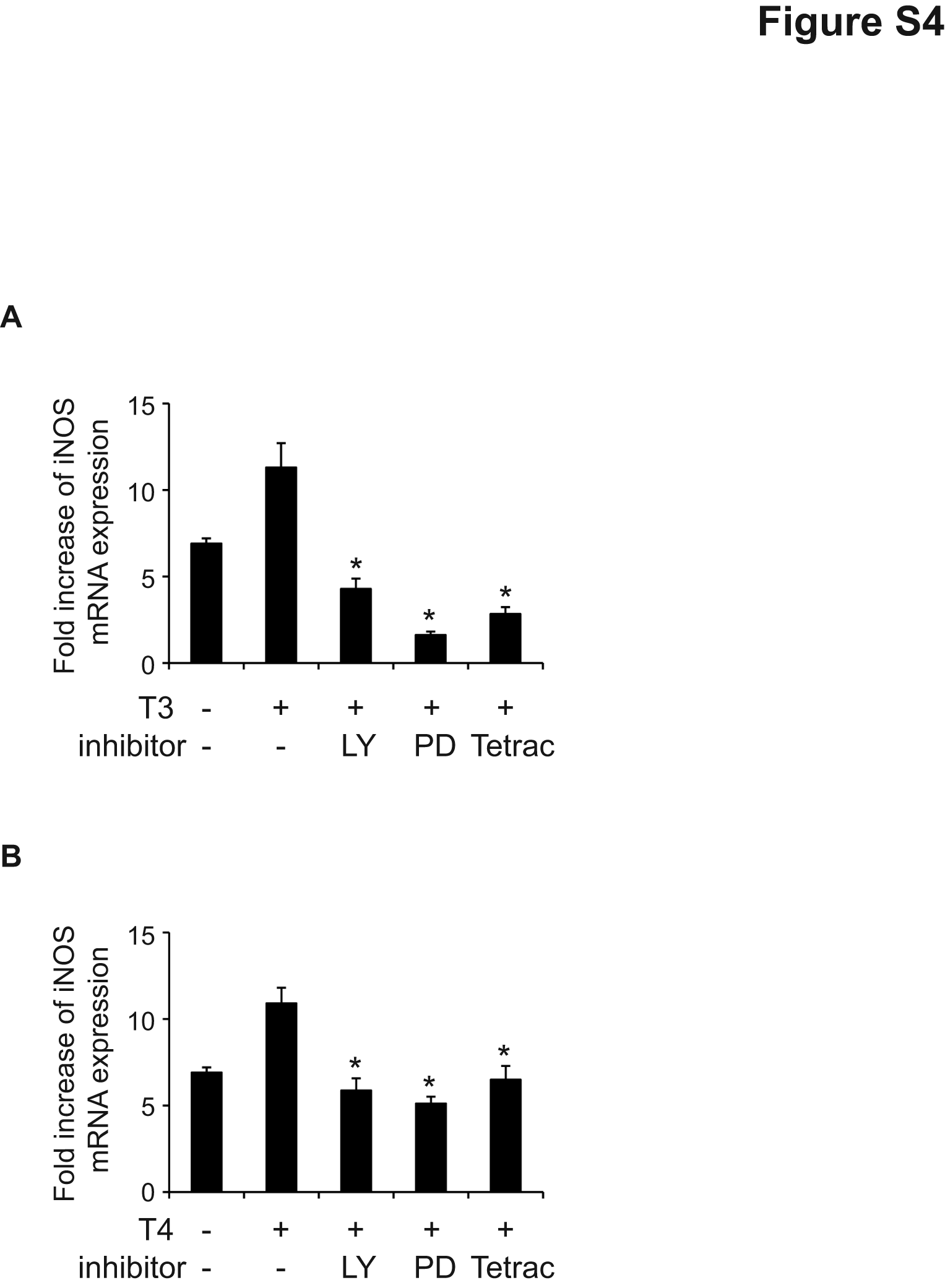

Supplement: Figure S4 — PI3K, ERK1/2 and integrin αvβ3 are involved in TH-enhanced iNOS expression. PMA-differentiated human THP-1 monocytes were treated with (A) 100 nM T3 or (B) 1 µM T4 for 24 h in the presence or absence of 2.5 µM LY294002 (LY, PI3K inhibitor), 10 µM PD98059 (PD, ERK1/2 inhibitor) or 10 µM tetraiodothyroacetic acid (Tetrac, T3/T4 analog to integrin αvβ3 receptor). Control cells were treated with vehicles (DMSO for inhibitors and NaOH for TH). Cells were infected with N. meningitidis FAM20 at a MOI of 200 for 24 h and the relative expression of iNOS was analyzed by real-time PCR. Data were normalized to the reference gene (RPL37A) and fold increase values compared to the uninfected condition are displayed. All experiments were performed in triplicate and results are presented as means ±SD. *, P<0.05 (Student’s t-test). (TIF) [file pone.0041445.s004.tif]
